# Proteomic analysis reveals key differences in pro-stromal corneal tissue between highly myopic males and females

**DOI:** 10.3389/fmed.2024.1406748

**Published:** 2024-08-16

**Authors:** Ge Cui, Yu Di, Shan Yang, Youxin Chen, Ying Li, Di Chen

**Affiliations:** ^1^Department of Ophthalmology, Peking Union Medical College Hospital, Chinese Academy of Medical Sciences & Peking Union Medical College, Beijing, China; ^2^Key Laboratory of Ocular Fundus Diseases, Chinese Academy of Medical Sciences & Peking Union Medical College, Beijing, China

**Keywords:** cornea, SMILE surgery, high myopia, sex difference, proteomic analysis

## Abstract

**Background and purpose:**

Nowadays, myopia has become a highly prevalent disease globally, especially in East Asia. Epidemiological studies have found that there may be sex differences in the occurrence and progression of myopia, with females having a higher incidence of myopia and higher risk of myopia progression. The purpose of this study was to explore the sex differences in myopic cornea using corneal stroma removed by small incision lenticule extraction (SMILE) surgery.

**Methods:**

The corneal stroma of females with high myopia (FH) and males with high myopia (MH) were subjected to proteomic assays. Proteomic-related data were statistically analyzed using software such as MaxQuan, KAAS, Proteome Discovery, etc. The total number of proteins in the cornea and the proteins specifically expressed in the two groups were counted, and the differentially expressed proteins in the two groups were identified by expression fold change >2 and *p*-value <0.05, and volcano plots were constructed, and functional enrichment analysis, subcellular organelle analysis, and molecular interaction were implemented.

**Results:**

Ten samples from each group were analyzed. Twenty-seven proteins were down-regulated and 27 proteins were up-regulated in the FH group, of which 23 proteins were up-regulated in the range of 2–10-fold and 4 proteins were up-regulated in the range of >10-fold. Comparative proteomic analysis of the cornea of male and female patients with high myopia revealed that the expression of corneal extracellular matrix and collagen I, III, V, and VIII-associated proteins were increased in the cornea of female patients, and the transforming growth factor-β (TGF-β)/Smad pathway was an important pathway obtained by functional analysis.

**Conclusion:**

Comparative proteomic analysis of cornea from male and female patients with high myopia revealed increased expression of proteins related to extracellular matrix and collagen I, III, V, and VIII in female patients, and the TGF-β/Smad pathway was an important pathway obtained from the functional analysis, suggesting that extracellular matrix remodeling and collagen fiber synthesis may be more active in the cornea of female patients.

## 1 Introduction

Nowadays, myopia is a highly prevalent disease worldwide, especially in East Asian. Some epidemiological studies (1–4) have found that there may be sex differences in the onset and progression of myopia, the prevalence of myopia and high myopia were higher in females, and the risk of myopia progression were greater in females than in males during the same observation time (5). There are many causes of myopia and its mechanisms are not yet clear. Some scholars have speculated that the sex differences in myopia may be related to behavioral patterns, because in adolescence, males tend to play more outdoor sports while females spend more time in close reading. The process of how behavioral patterns affect the ocular anatomy and its mechanisms need to be investigated. Therefore, the purpose of this study was to explore the proteomic differences in myopic cornea of different sexes using corneal stroma removed during small incision lenticule extraction (SMILE) surgery.

Human corneal tissue is divided into five layers, among which the stromal layer is the thickest, accounting for about 90% of the total corneal thickness (6). The stromal layer provides a major part of the refractive power of the whole corneal tissue and keeps the cornea completely transparent due to its highly orderly arrangement and hydrophobic properties. The corneal stroma is composed of multiple layers of uniformly arranged fine collagen fibers with corneal stromal cells scattered between the fibers (7). The extracellular matrix (ECM) of corneal stromal layer is composed of 1,679 different proteins (8). The main components of corneal stroma are type I collagen and Type V collagen, among which type V collagen can promote the formation and maintenance of stromal microfiber structure (8). Other main components of corneal ECM are proteoglycan, glycoprotein, etc. (9). The properties and functions of these complex protein and fibrous components are inconclusive (10), and their synthesis, assembly, degradation, and remodeling in the stromal layer of the cornea in highly myopic patients have not been thoroughly investigated yet.

ECM is a complex three-dimensional network of macromolecular structures (11) such as collagen, proteoglycan, glycoprotein and elastin, which can provide structural and biochemical support for surrounding cells. ECM also regulate intracellular communication and influence cellular behavior, regulating gene expression and a variety of functional properties through its interactions between cell surface receptors. ECM not only serve as a solid support for cells, but also act as a reservoir for a variety of essential cytokines and growth factors (12), such as vascular endothelial growth factor (VEGF), transforming growth factor (TGF) and protease (13, 14), thus providing a library of bioactive molecules that contribute to tumor cell metastasis and angiogenesis. The key component of ECM is proteoglycan (PGs), which is composed of core proteins. Remodeling of PG in tumor ECMs and cell membranes could affect cancer cell properties, such as cell proliferation, migration, invasion, angiogenesis and adhesion (15). The most richly expressed PG in ECM are small leucine-rich PGs (SLRPs), which influence the regulation of key cell functional features, namely, cancer cell migration, autophagy, angiogenesis, and metastasis potential (16).

Estrogen has an antifibrotic effect. After binding with ERα, estrogen may have a protective effect on some fibrosis diseases, as shown by inhibiting TGF-β/Smad signaling pathway in the treatment of liver fibrosis, diabetic nephropathy and fibrocavernositis (17, 18), inhibiting renal interstitial fibroblast activation and atherosclerosis (19), it also prevents ischemic myocardial injury and myocardial fibrosis (20). Animal studies have shown that left ventricular volume overload induces a protective increase in ERβ mediated by TGF-β-induced protein IG-H3 by inhibiting neutrophil adhesion to the endothelium, but this protective compensated effect disappears after ovariectomy. 17-β estradiol limits cardiac hypertrophy caused by chronic volume overload and improves cardiac function in ovariectomized rats. Estrogen limits poor ECM remodeling and left ventricular dilatation (21), in part by regulating ECM protein expression in the heart, this cardioprotective mechanism may involve the interaction between estrogen, ER, and ECM. Ovarian hormone deficiency is associated with decreased ERα expression, suggesting that ERα deficiency may also contribute to ECM remodeling during the progression of volume overload to heart failure (21). Estrogen may contribute to ECM synthesis and degradation, and postmenopausal estrogen deficiency may cause ECM turnover disorders, leading to basal membrane abnormalities, Bruch’s membrane thickening, and sediment accumulation under RPE in age-related macular degeneration (ARMD).

Estrogen can automatically regulate ER, and only physiological concentration of estrogen can induce ER expression and increase the activity of matrix metalloproteinase (MMP-2). ER subtypes are co-expressed at different levels and mediate different cell functions. Dose-dependent differences in activation and expression of ERα and ERβ may induce complex downstream interactions that lead to negative feedback on ER expression. Estrogen regulates MMP-2 activity and protein expression through the NF-κB pathway. The NF-κB inhibitor (22), can inhibit the regulation of MMP-2 by estrogen. Since estrogen and NF-κB are antagonistic in some systems, high levels of estrogen may significantly up-regulate NF-κB. This results in contradictory inhibition of ER signal transduction. Abnormal expression of MMP is associated with the progression of fibrosis related diseases.

Due to the small sample size of the corneal pro-stroma collection, highly sensitive, repeatable and reliable detection methods are required. Proteomics is important for exploring the biomarkers and pathogenesis behind the disease (23). Previous proteomic studies on human corneal tissue (24) provide insight for the study of keratoconus (25), Fuch’s corneal endothelial dystrophy (26) and diabetes (27). Compared with immunoassay, this method has the advantages of improving reproducibility, accuracy and sensitivity. Proteomic and functional analysis of human cornea may help elucidate the unknown biological functions of proteins and explore pathogenesis at the molecular level. In the past, there were limited research on the pathogenesis of myopia using proteomics. This study aimed to explore the proteomic information of the pro-stroma obtained from SMILE surgery on myopic patients through non-targeted mass spectrometry (MS) proteomics, to better understand the molecular mechanism of sex differences in myopic cornea.

## 2 Materials and methods

### 2.1 Ethical approval

The ethical committee of Peking Union Medical College Hospital (PUMCH) authorized all of the study’s experiments, which were conducted in accordance with the Declaration of Helsinki (ZS-3516). Prior to the collection of tissues, written and informed consent was obtained. All procedures were carried out in accordance with institutional and governmental regulations, and all human samples were de-identified before analysis.

### 2.2 Corneal stroma collection

**Figure 1:**
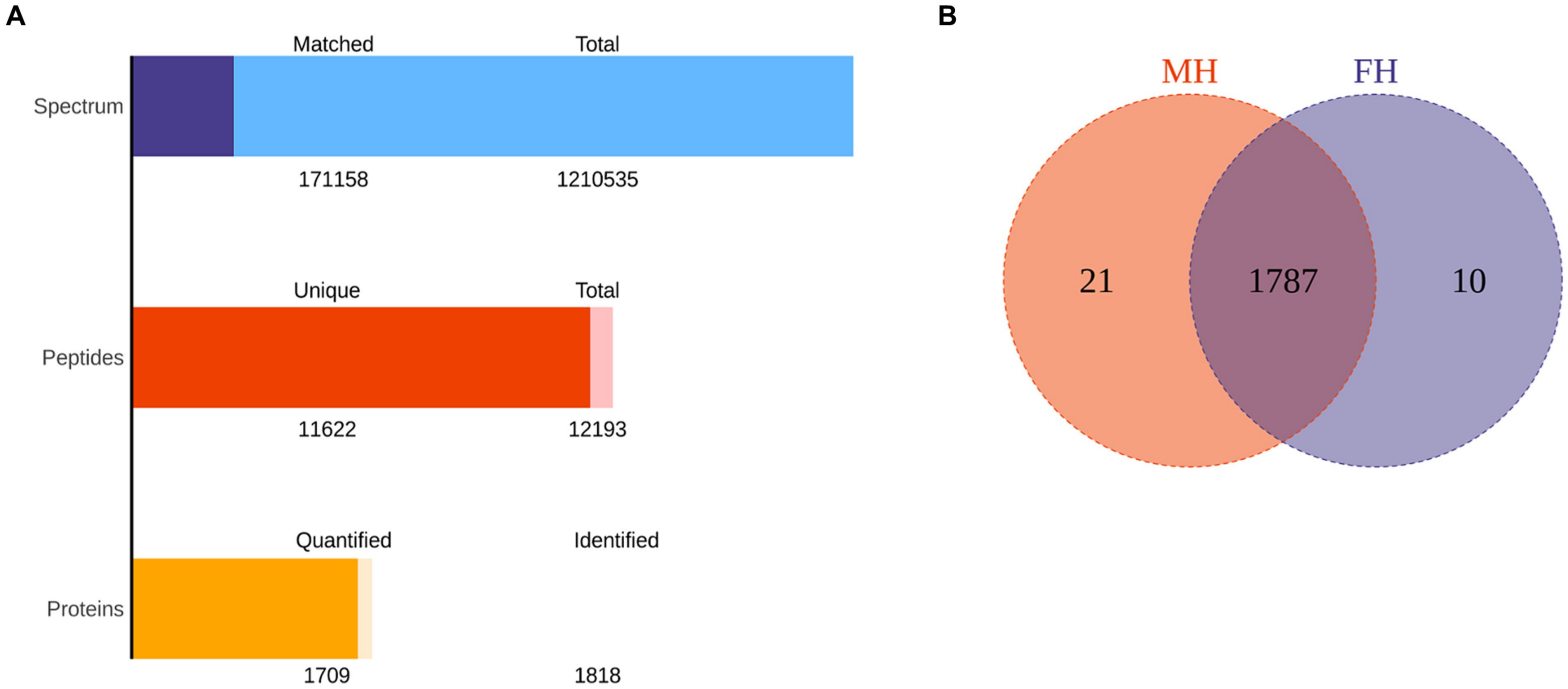
The total protein and peptide of MH group and FH group were identified by proteomics. **(A)** The quantity of total protein and peptide of two groups. **(B)** The number of common and differentially expressed proteins in two groups.

Complete general and ophthalmic histories were collected from all participants. Patients with history of active ocular diseases, systemic conditions, or taking systemic medications were excluded from the study. Patients with high myopia that sphere equivalent (SE) of both eyes >−6.00 D who underwent SMILE surgery in the Department Ophthalmology of PUMCH from August 2020 to April 2023 were included. The patients were age-matched and range from 18 to 35 years old and were divided to 2 groups: females with high myopia (FH) and males with high myopia (MH), 10 samples in each group (Figure 1A). Before surgery, a comprehensive ophthalmic examination was performed, including manifest refraction, cycloplegic refraction (KR- 3500, Topcon, Tokyo, Japan), slit-lamp bio-microscopy, corneal topography (Tomey TMS-4; Tomey, Nagoya, Japan), central corneal thickness (AL-3000; Tomey, Nagoya, Japan), axial length (IOL master 700, Carl Zeiss Meditec, Jena, Germany) and dilated fundus examination.

The corneal pro-stroma sample was obtained by routine operation, cleaned by sterile PBS, placed into sterile and enzyme-free storage tube with thread, and then quickly transported in liquid nitrogen. The sample were stored at −80°C (less than 1 month) before they were tested for proteomic detection.

### 2.3 Protein extraction and peptide enzymolysis

Samples were ground into coarse powder in liquid nitrogen, and protein was extracted by SDT cracking method, using 4% (w/v) SDS, 100 mM Tris/Hcl and 0.1 M DTT at PH 7.6. The cracking materials were centrifuged at 14,000 × *g*, 4°C for 10 min, then the protein concentration in the supernatant was determined (BCA method). As previously reported (28, 29), 180 μg of the above corneal protein lysates were extracted from each sample, reduced with 10 mM dithiothreitol at 37°C for 60 min, alkylated with 20 mM iodoacetamide at 37°C for 30 min (darkroom), and precipitated with 250 μL 100% ice acetone at −20°C for 12 h, and centrifuged at 14,000 × *g*, 4°C for 10 min. The precipitates were suspended in 50 mM TEAB until the final protein concentration was 1 μg/μl, and then digested by trypsin at 1:50 (w/w) enzyme/substrate ratio in a 37°C-incubator shaker for 16 h. The digestion was terminated by adding 10% formic acid to the final concentration of 0.1%, and the peptide was demineralized using C18 column. The rinsed solution was removed and dried using a vacuum concentrator. The lyophilized samples were stored at −20°C and redissolved with 40 μL 0.1% formic acid solution. The peptides were quantified at 280 nm absorbance.

### 2.4 LC–MS/MS data collection

Each sample was separated by the liquid phase system Easy nLC using high performance liquid chromatography (HPLC) at nanolit-rate, the lyophilized peptides were extracted, and the peptides were separated by high PH 2-D on the column, concentrated into 80 fractions, and combined into 40 fractions. The distillate was lyophilized and the buffer solution (28) was used on the loading column at a flow rate of 300 nL/min and separated through the analytical column. The samples were separated by chromatography and then analyzed by mass spectrometry. All samples were obtained using trapped ion mobility spectrometry with Captive Spray nano electrospray ion source time-of-flight mass spectrometer (TIMS-TOF Pro, Bruker Daltonics). The ion source voltage was set at 1.5 kV, the detection mode was positive ion, the mass spectrum scanning range was set at 100–1700 m/z, and the charge number was in the range of 0–5.

### 2.5 Protein identification and quantitative analysis

Identification and quantitative analysis were performed using MaxQuant software (version 1.6.14) (30). This study focused on the differences between FH and MH groups, so the protein quantitative method was LFQ method, which is a relative quantitative method for pairwise comparison between multiple groups, corrected by pair-wise of peptide and protein layers (31). Corresponding to this method is iBAQ (Intensity-based absolute quantification), which is used for the absolute quantification of protein samples. The main algorithm is based on the ratio of the sum of the identified peptide intensity of the protein to the theoretical number of peptide segments (32).

### 2.6 Statistical analysis

SPSS 23.0 was used to compare demographic and clinical data between the two groups, and *p* < 0.05 was considered statistically significant. Two independent sample *T*-test was used for normal data, and the original LC–MS/MS file was analyzed using online Peaks (Bioinformatics Solutions Inc.) and Proteome Discovery software (version v.4.1). The data were retrieved from the UniProt human database, and a false discovery rate (FDR) <1% for proteins and peptides was set as the threshold for protein identification. The retrieved protein peak area was used for subsequent statistical analysis. Local polynomial fits for the number of proteins in each group and the rate of protein detection were calculated using locally weighted polynomial regression (lowess package in R v.0.3.50) to estimate proteomic data. Proteins with a total deletion value of >50% were removed and the minimum area in the group was used to fill the remaining blank value. For the discovery of biomarkers, the expression factor (Fold Change, FC) and *p*-value (*T*-test or other) were used as the criteria, and the protein with FC > 2 (up-regulated greater than 2 times or down-regulated less than 0.50) and *p* < 0.05 were considered as differentially expressed proteins. The Gene Ontology (GO) analysis and KEGG pathway enrichment analysis were performed using Fisher’s exact test. Significantly enriched pathways contained at least three genes and *p*-values adjusted for Benjamini-Hochberg <0.05. STRING database was used to construct the protein interaction network of differentially expressed proteins, with confidence >0.9 as the critical level.

## 3 Results

### 3.1 Clinical data characteristics

Except for sex, the other clinical parameters of the two groups were equally comparable. The age of the FH group was 26.40 ± 2.06 years old and the SE was −6.95 ± 0.69 D, and the age of the MH group was 26.70 ± 3.70 years old and the SE was −6.99 ± 0.96 D (Table 1).

**Table 1:** Comparison of preoperative and intraoperative clinical parameters of FH and MH groups.

	MH group (*n* = 10)	FH group (*n* = 10)	*p*-value
Age	26.40 ± 2.06	26.70 ± 3.70	0.078
Preoperative			
Sphere	−6.39 ± 0.80	−6.46 ± 1.00	0.288
Cylinder	−1.18 ± 0.77	−1.18 ± 0.78	0.908
SE	−6.95 ± 0.69	−6.99 ± 0.96	0.059
CDVA	1.05 ± 0.89	1.05 ± 0.09	0.864
CCT	557.75 ± 22.01	565.68 ± 25.82	0.153
K1	43.09 ± 0.53	43.11 ± 1.65	0.224
K2	42.77 ± 1.10	42.86 ± 1.49	0.815
Intraoperative			
Min thickness	14.83 ± 0.56	14.72 ± 1.18	0.337
Max thickness	135.25 ± 3.30	134.11 ± 6.78	0.098
Optical zone	6.40 ± 0.12	6.40 ± 0.10	0.859

### 3.2 Identification quantitative analysis

The data of FH and MH groups were analyzed and compared by proteomics, as shown in Figure 1. One thousand eight hundred eighteen proteins were identified in all samples, and 1709 proteins were quantified. The total number of peptides was 12,193, and the total number of unique peptides was 11,622. There were 1,210,535 secondary spectral maps and 171,158 database matched spectral maps. The protein identification quantities of 10 samples in FH group were 1,575, 1,479, 1,356, 1,565, 1,410, 1,601, 1,488, 1,268, 1704, 1,578, respectively. The identified protein quantities of 10 samples in MH group were 1,645, 1,604, 1,655, 1,692, 1705, 1,403, 1,420, 1,699, 1,627 and 1,662, respectively. In all samples, the top 20 most abundant proteins identified in the corneal stroma are shown in Table 2. There were 10 and 21 specific proteins in the FH and MH groups, respectively, and 1787 proteins in common between the two groups (Figure 1B). The specific proteins in FH group were P05062, P10620, P16422, P40306, P55327, Q02790, Q14139, Q15493, Q96PP8, and Q9H0F5. The proteins specific to MH group are: O43493, O95050, P02810, P16455, P20062, P30047, P42566, P98088, Q13435, Q14508, Q14746, Q2M389, Q53RT3, Q562R1, Q86X83, Q8IY95, Q8TEA8, Q96KA5, Q9Y6I3, Q9NP58, and Q9NZH6.

**Table 2:** Top 20 proteins identified by corneal pro-stromal proteomics.

	Protein names	Gene names
1	Collagen alpha-3 (VI) chain	COL6A3
2	TGF-β-inducing protein IG-H3	TGFBI
3	Collagen alpha-1 (VI) chain	COL6A1
4	Collagen alpha-1 (XII) chain	COL12A1
5	Collagen alpha-2 (VI) chain	COL6A2
6	Lumican	LUM
7	Immunoglobulin gamma-1 heavy chain	
8	Corneal protein	KERA
9	Decorative protein	DCN
10	Contains protein 2 of the MAM domain	MAMDC2
11	disaccharide	BGN
12	Acetaldehyde dehydrogenase, dimer NADP primary type	ALDH3A1
13	Platelet reactive protein-4	THBS4
14	Immunoglobulin κlight chain	
15	Collagen alpha-1 (I) chain	COL1A1
16	Vimentin	VIM
17	Immunoglobulin weight constant gamma 2	IGHG2
18	Recombinant human annexin A2 (annexin)	ANXA2
19	Angiopoietin associated protein 7	ANGPTL7
20	Complement C3	C3

### 3.3 Expression difference analysis

**Figure 2:**
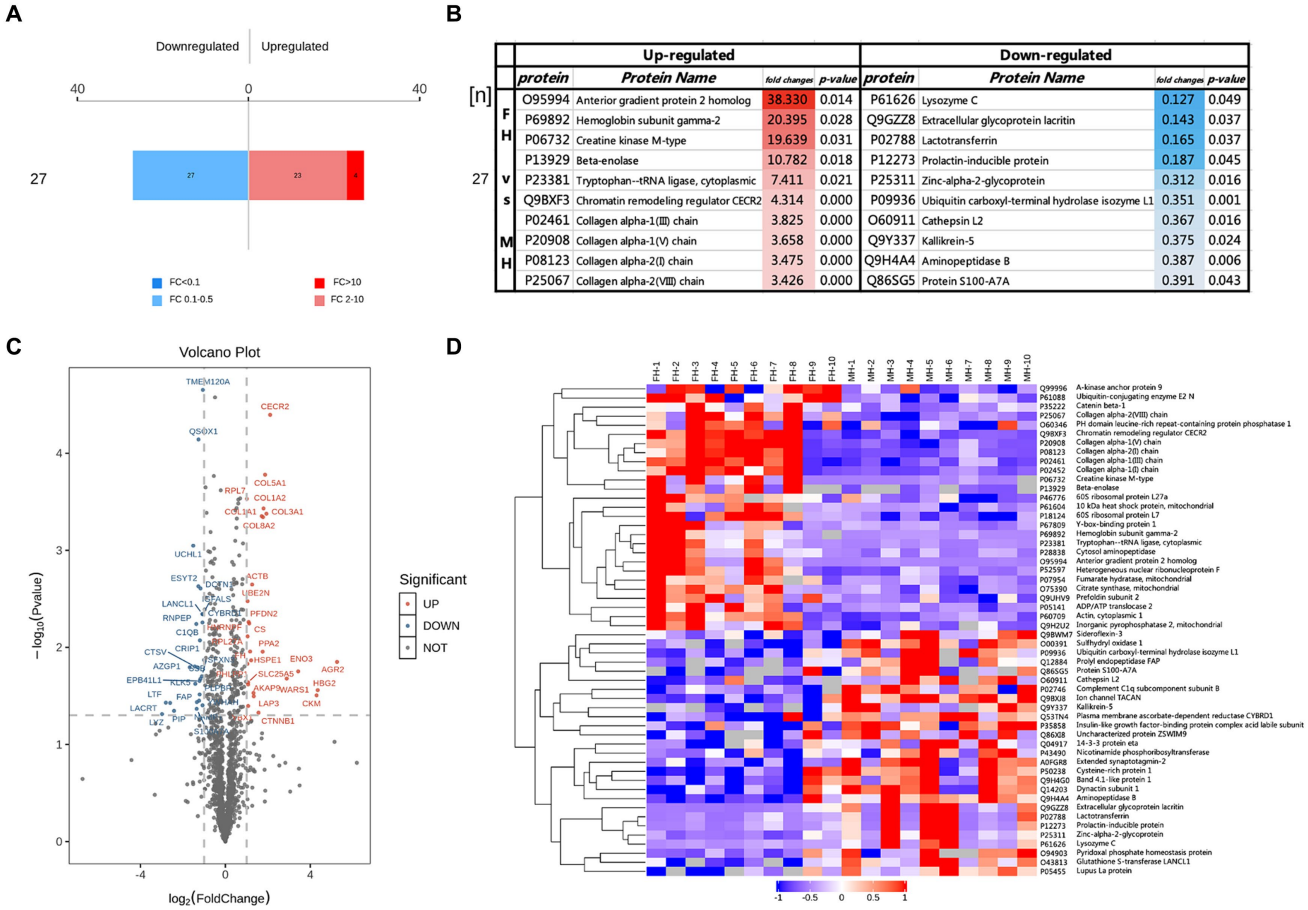
Different proteins in FH group compared with MH group, **(A)** FC > 2 and *p* < 0.05 represented up-regulated, shown in red; FC < 0.50 and *p* < 0.05 represented down-regulated, shown in blue. **(B)** The top10 up-regulated and down-regulated proteins in FH group compared with MH group. **(C,D)** Volcano maps and heat maps of the different proteins in two groups.

FC > 2 and *p* < 0.05 were used as the criteria to screen differential proteins, and the up-regulated and down-regulated protein numbers between FH and MH groups were obtained (Figure 2), the protein which up-regulated >10 were screened. Furthermore, FC and *p*-values were used to draw volcano maps and heat maps (Figures 2C,D), in which the proteins significantly up-regulated (FC > 2 and *p* < 0.05) were labeled as red dots or strips, while those significantly down-regulated (FC < 0.5 and *p* < 0.05) were labeled as blue dots or strips, undifferentiated proteins were gray dots or strips.

Twenty-seven down-regulated proteins and 27 up-regulated proteins were found in the FH group compared with the MH group, among which 23 proteins were up-regulated by a FC of 2–10 (Figure 2A) and 4 proteins were up-regulated by a FC >10 (Figure 2B). The four proteins were anterior gradient protein 2 homolog (O95994), hemoglobin subunit γ2 (P69892), creatine kinase M-type (P06732), and *β*-enolase (P13929). Of the remaining up-regulated proteins, COL1A1, COL1A2, COL3A1, COL5A1, COL8A2, and fibroblast activating protein (FAP) form a tight interaction network, and the expressions of four types of collagen were significantly up-regulated: type I collagen, type III collagen, type V collagen and type VIII collagen, in addition to tryptophan-tRNA ligase, chromatin remodeling regulator CECR2 expression upregulated. The most significantly down-regulated proteins were prolactin-inducible protein, lactoferrin, acritin and lysozyme C.

### 3.4 Functional analysis

**Figure 3:**
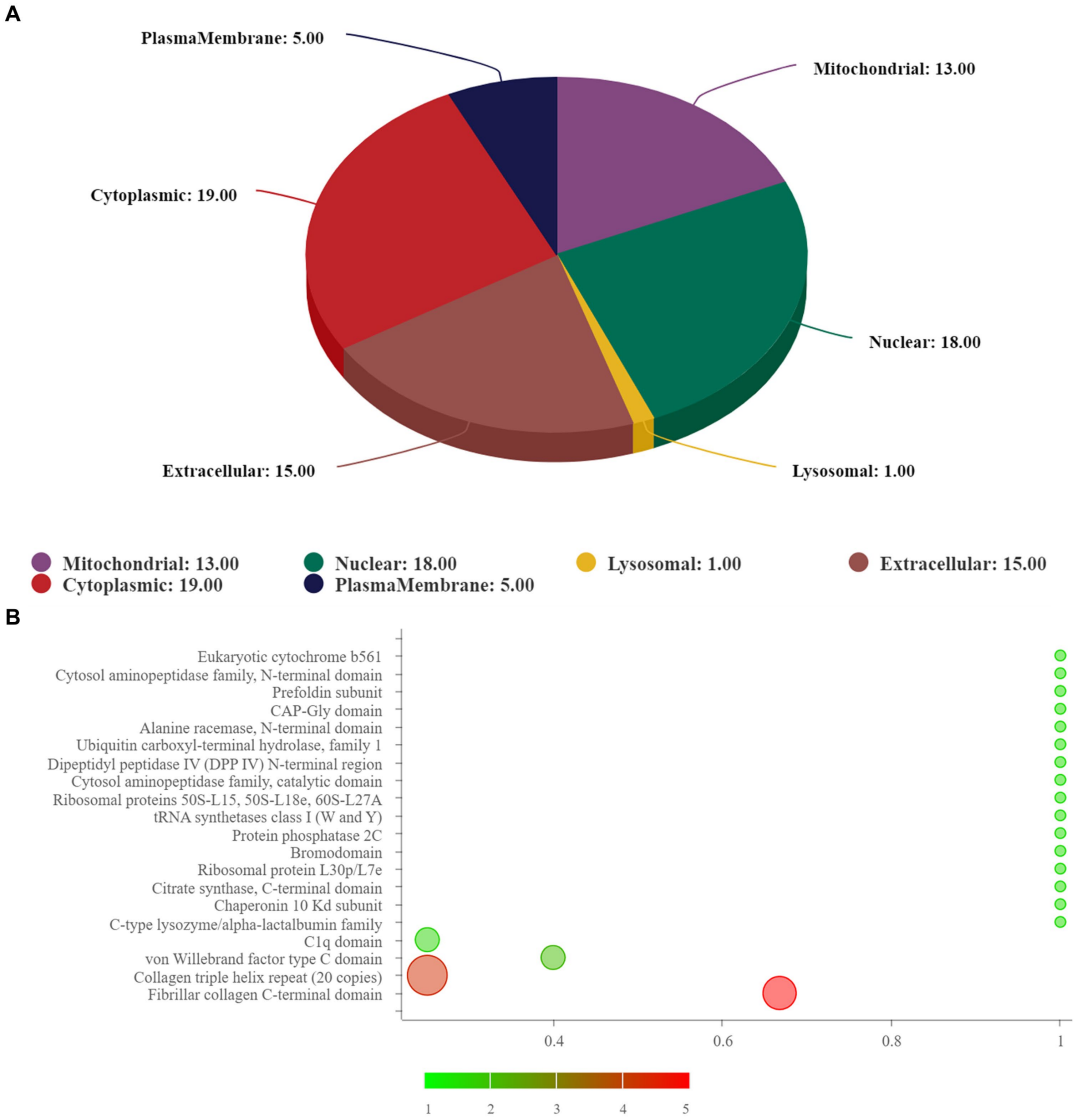
**(A)** Subcellular localization analysis; **(B)** prediction of domain. In **(B)**, the abscissa represents rich factor (value ≤1), the ordinate represents the statistical results of different proteins in each domain classification. The redder the bubble color, the smaller the *p* value (−log10), that means the higher the enrichment significance level in the corresponding domain classification. The bubble size represents the amount of protein.

The subcellular structure prediction software CELLO and domain prediction software interproscan were used to perform subcellular localization analysis and domain prediction for all differentially expressed proteins of the two groups, and the number of proteins in each subcellular localization and domain was shown in Figure 3A. The two most significant domain enrichment analyses were fibrillar collagen C-terminal domain and collagen triple helix repeat (20 copies) (Figure 3B), and the domain with high enrichment content was: collagen triple helix repeat (20 copies), fibrillar collagen C-terminal domain, von-Willebrand factor C-type domain, C1q domain, leucine rich repeat (LRR) and RNA recognition motif (also known as RRM, RBD or RNP domain).

### 3.5 Go analysis

**Figure 4:**
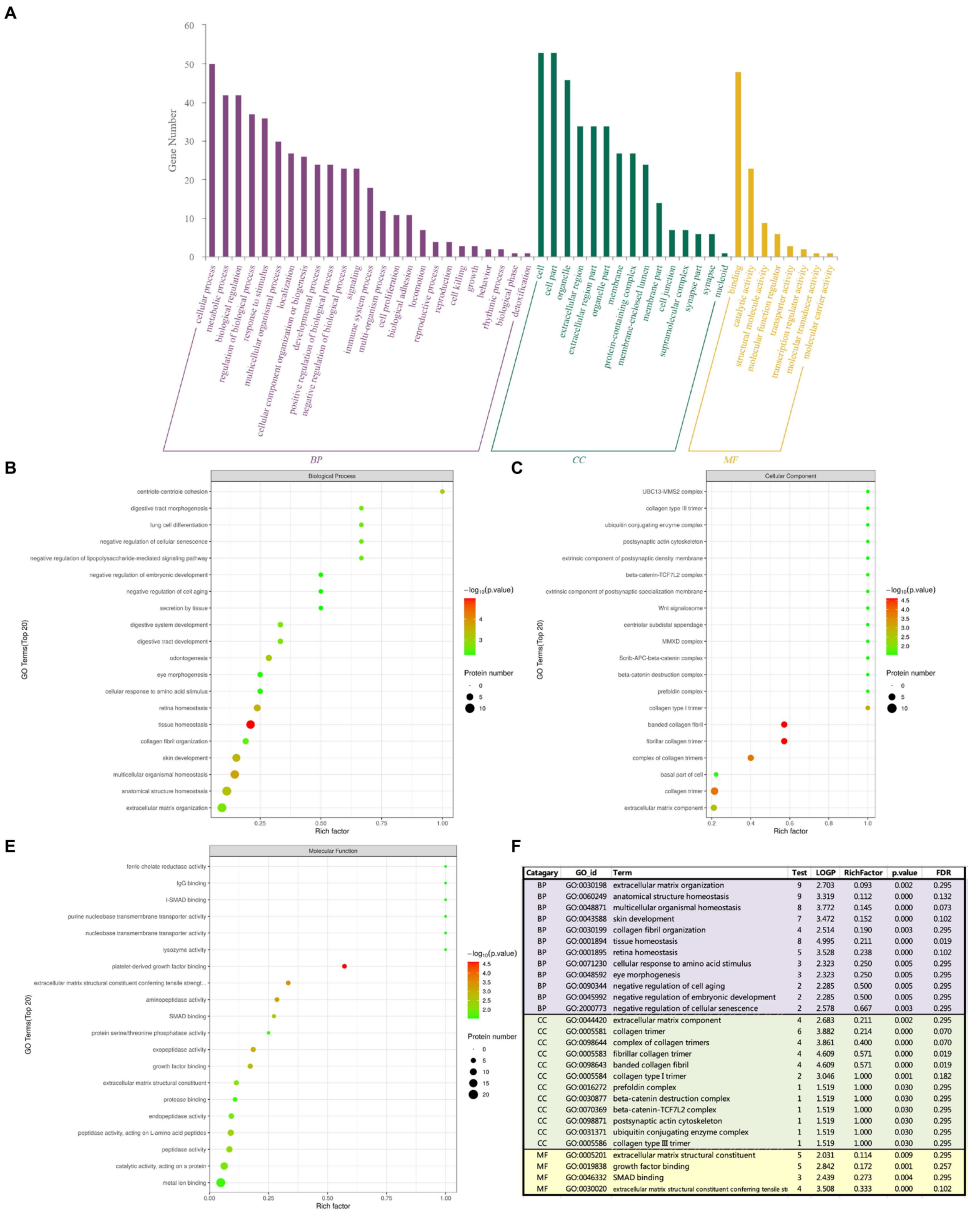
The abscissa represents enrichment factor, that is the number of differentially expressed proteins classified as a GO sub-functional category/the total number of proteins identified in this category (value ≤1); the ordinate represents the different protein expression results of each sub-functional category; The redder the bubble color, the smaller the p value (−log10), that means the higher the enrichment significance level in the corresponding sub-functional categories. The bubble size represents the amount of protein. **(A)** Total GO analysis; **(B)** BP analysis; **(C)** CC analysis; **(D)** MF analysis; **(E)** Summary analysis.

The GO analysis includes the following three items: biological process (BP), cellular component (CC) and molecular function (MF), the number of different proteins obtained by analysis was positively correlated with the importance of corresponding functional classes. As shown in Figure 4, GO enrichment highlighted the top 5: tissue homeostasis, platelet-derived growth factor binding, fibrillar collagen trimer, banded collagen fibril, and collagen trimer. GO enrichment analysis showed that BP had collagen fibrous tissue, retinal homeostasis, extracellular matrix, ocular morphogenesis, cell aging and embryonic development. CC has extracellular matrix components, collagen trimers and their complexes, banded collagen fibers, type I and type III collagen trimers, postsynaptic actin cytoskeleton and *β*-catenin-related complexes, and MF has extracellular matrix that gives tensile strength and Smad protein binding. Tissue homeostasis was the most significant in BP enrichment analysis; in CC enrichment analysis, the banded collagen fibril, fibrillar collagen trimer, collagen trimer and complex of collagen trimers were the most significant; and platelet derived growth factor binding was the most significant in MF enrichment analysis. Combined with the analysis of *p*-value and enrichment factor, CC enrichment was closely related to extracellular matrix and collagen components: extracellular matrix components, the basal part of cells, collagen trimer, complex of collagen trimers, fibrillar collagen trimer, banded collagen fibril, type I collagen trimer, *β*-catenin.

### 3.6 KEGG enrichment analysis

**Figure 5:**
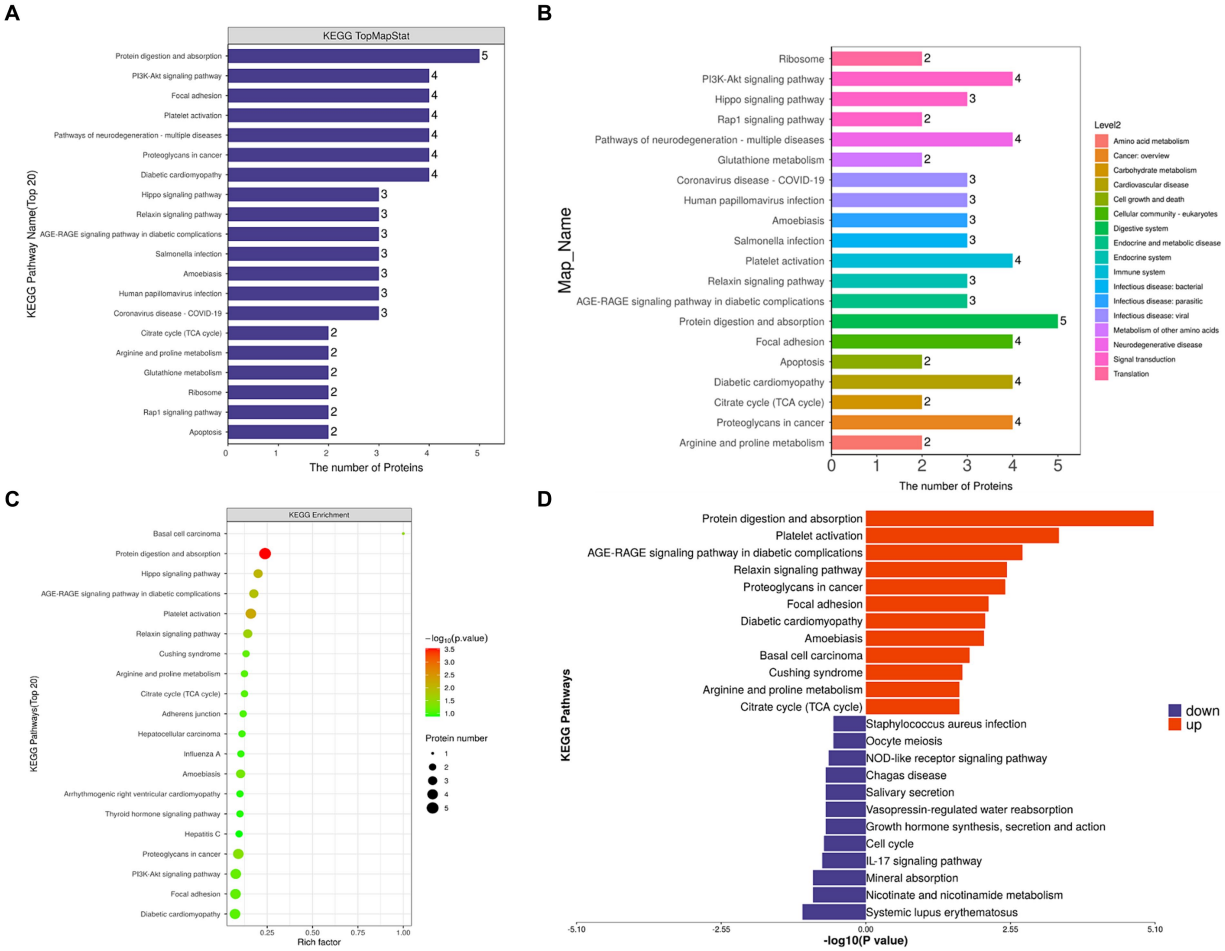
**(A,C)** KEGG analysis shows the signaling pathway with the participation of different proteins. The ordinate represents the name of signaling pathway, and the abscissa represents the number of different proteins contained in the pathway. The more proteins, the more important the pathway is. **(B)** Different colors represent the seven branches of KEGG’s metabolic pathway. **(D)** Up-regulated and down-regulated KEGG pathways in FH group compared with MH group.

KEGG pathway annotation and number statistics were performed for all differentially expressed proteins in FH and MH groups, as shown in Figure 5. The top 7 pathways of KEGG pathway enrichment include protein digestion and absorption, platelet activation, Hippo signaling pathway, AGE-RAGE signaling pathway in diabetes complications, relaxin signaling pathway, basal cell carcinoma, and proteoglycan in cancer. The KEGG metabolic pathway is shown in seven branches as follows: cellular processes, environmental information processing, genetic information processing, human diseases (animals only), metabolism, organic systems, and drug development.

By comparing the KEGG results of differential proteins with those of all identified proteins, a significantly enriched (*p* < 0.05) KEGG metabolic pathway was obtained (Figure 5): protein digestion and absorption, platelet activation, proteoglycan in cancer, PI3K-Akt signaling pathway, local adhesion, diabetic cardiomyopathy, Hippo signaling pathway, AGE-RAGE signaling pathway in diabetic complications, relaxin signaling pathway, amoebiasis, Cushing’s syndrome, citrate acid cycle (TCA cycle), arginine and proline metabolism, adhesion nodes, hepatocellular carcinoma, influenza A, thyroid hormone signaling pathway, arrhythmogenic right ventricular cardiomyopathy, hepatitis C. The up-regulated and down-regulated differential proteins were separated for enrichment analysis of the KEGG pathway, and the up-regulated proteins were involved in the following pathways (Figure 5D): protein digestion and absorption, platelet activation, the role of AGE-RAGE signaling pathway in diabetic complications, relaxin signaling pathway, proteoglycan in cancer, adhesion nodes, diabetic cardiomyopathy, amoebiasis, Cushing’s syndrome, basal cell carcinoma, arginine and proline metabolism, citrate acid cycle (TCA cycle); down-regulated proteins were involved in the following pathways: staphylococcus aureus infection, oocyte meiosis, Nod-like receptor signaling pathway, American Trypanosomiasis, saliva secretion, vasopressin-regulated water reabsorption, growth hormone synthesis secretion and action, cell cycle, IL-17 signaling pathway, mineral absorption, nicotinate and nicotinamide metabolism, and systemic lupus erythematosus.

### 3.7 Protein–protein interaction

**Figure 6:**
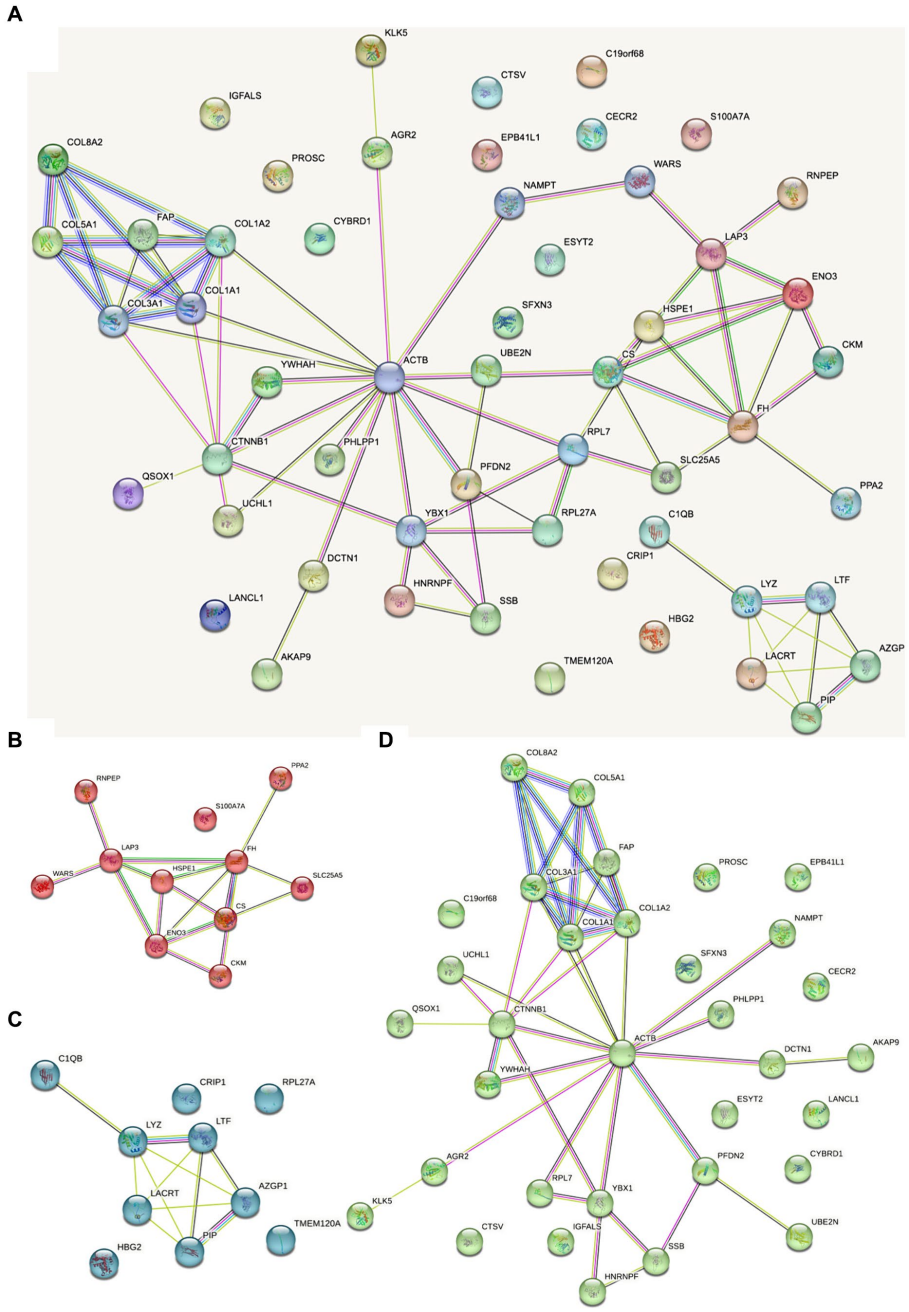
Dots represent different proteins, and the colors of dots represent different clusters. The lines represent the interactions between proteins, and the thickness of the lines between two proteins represents the intensity of their interactions. The proteins that interact with more other proteins need more focus. **(A)** Total protein–protein interaction; **(B)** the cluster mainly composed of carboxylic acid metabolic process and mitochondrial matrix; **(C)** the cluster mainly composed of retinal and tissue homeostasis; **(D)** the cluster mainly composed of collagen fiber tissue and extracellular matrix tissue.

The protein interaction map of the different proteins of FH group and MH group was drawn on the STRING website (Figure 6). As shown in the figure, actin located in cytoplasm was the most closely related to other proteins. Actin is highly conserved, widely expressed in all eukaryotic cells, and is associated with various types of cell movement. Based on the principle of topological structure recognition, the proteins with high aggregation degree in the interaction network diagram were divided into three different clusters: the cluster mainly composed of collagen fiber tissue and extracellular matrix tissue, the cluster mainly composed of retinal and tissue homeostasis, and the cluster mainly composed of carboxylic acid metabolic process and mitochondrial matrix.

## 4 Discussion

In this study, proteomic detection was carried out on the corneal lenticules from highly myopic females and highly myopic males. A total of 1818 proteins were identified and 1709 proteins were quantified. Fifty-four differentially expressed proteins were identified in the two groups using fold change >2 and *p* < 0.05 as criteria. It was found that the expression of corneal extracellular matrix and collagen I, III, V, and VIII-related proteins were increased in female patients, as well as the expression of TGF-β and Smad protein-related signaling pathways, suggesting that corneal extracellular matrix remodeling and collagen fiber synthesis may be more active in female patients, and thus the changes in the cornea of female patients may contribute to the onset and progression of myopia. In addition, Smad protein, as a downstream protein of TGF-β, may also be involved in the remodeling of the extracellular matrix and influence the development of myopia.

Studies have shown that SMILE lenticules were histologically consistent with the *in vivo* cornea (33, 34): corneal stromal cells were scattered between the dense extracellular matrix. Examination of SMILE lenticule at the histological level by the Sirius scarlet staining revealed that the main components of corneal stromal cells was collagen, especially mature type I collagen fiber (34), but no reticular fiber or elastic fiber (35) was found. Protein components that play an important role in the structural and functional stability of collagen fibers, such as proteoglycans, glycoproteins and crystallized proteins, were present in SMILE lenticule (6). As the main components of ECM, these protein components can now be identified and quantified (34). Type V collagen accounts for 10–20% (36) of corneal collagen. Type V collagen forms heterogeneous collagen fibers with type I collagen, which is crucial for the assembly of fibers and the normal structure of corneal stroma (37). Gene mutation of type V collagen can lead to the disarrangement of corneal collagen fiber layers, making cornea opaque, and thus affecting the normal function of cornea (38).

The most abundant protein in the anterior corneal stroma of high myopia was type VI collagen, which ranks among the top 5 in the terms of the content of collagen 1, 2, and 3 chains, and there were also high levels of type I collagen, type XII collagen, TGF-β-inducible protein IG-H3, lumican, keratocan, decorin, vimentin, complement C3, immunoglobulin. Proteins that were up-regulated more than 10-fold in the FH group relative to the MH group included: anterior gradient protein 2 homologs, which may play a role in cell migration, cell differentiation, and cell growth and promote cell adhesion; hemoglobin subunit γ2, which is a component of fetal hemoglobin; creatine kinase type M, which promotes the energy transduction of skeletal muscle and heart; and *β*-enolase, which promotes the development and regeneration of transverse striated muscle. Among other upregulated proteins, which include four collagens: type I collagen, type III collagen, type V collagen and type VIII collagen. Type I collagen is a member of group I collagen (fibrillar-forming collagen), type III and type I collagen are widely found in connective tissue. Type VIII collagen macromolecule is a major component of the basement membrane of corneal endothelial cells and vascular endothelium. α2 chain of type VIII collagen is encoded by the COL8A2 gene, defect of this gene is associated with Fuch’s endothelial corneal dystrophy and posterior polymorphic corneal dystrophy type 2, and its related pathways include collagen chain trimerization and integrin pathway, with GO annotations related to this gene include ECM structural components and protein-macromolecular bridging subunit activity.

TGF-β-inducible protein IG-H3, the second most abundant protein in corneal stroma, is encoded by the βIG-H3 gene, which was first identified by differential screening of cDNA libraries extracted from A549 human lung adenocarcinoma cells treated with TGF-β and was located on human chromosome 5q31. TGF-β-inducer protein IG-H3 contains 683 amino acids, with a secretory signaling sequence and four homologous internal domains, commonly seen in hereditary corneal dystrophy, characterized by corneal amyloid deposition, which plays a role in cell adhesion and may influence cell-collagen interactions (39). It is involved in angiogenesis, cell adhesion, cell proliferation, ECM structural components, collagen binding and other GO pathways. It can increase the synthesis and secretion of collagen and fibronectin, accelerate wound healing, stimulate the production of basic fibroblast growth factor-binding proteoglycan, and regulate the phosphorylation of epidermal growth factor receptor.

Previous studies have shown that platelets up-regulate the expression of StAR, HSD3B2, aromatase and HSD17B1 genes by activating the NF-κB and TGF-β/Smad pathways, thus promoting the secretion of estradiol in endometrial stromal cells (increased by about 4.5 times) (40). As an important pathway related to ECM remodeling, TGF-β/Smad also exists in the corneal stroma (41). In this study, the main proteins involved in TGF-β/Smad pathway were identified as follows: polyligand glycan-binding protein 1, endothelial glycoprotein, skin bridging protein, CD109 antigen. Polyligand glycan-binding protein 1 (syntenin-1): positively regulates TGF-β-mediated Smad activation and TGF-β-induced epithelial interstitial transformation (EMT) and cell migration in various cell types. It is possible to increase TGF-β signaling by enhancing the cell surface expression of TGFR1 by preventing the interaction between TGFR1 and CAV1 and subsequent CAV1-dependent internalization and degradation of TGFR1 (42). Vascular endoglin (43) plays an important role in the regulation of angiogenesis, it regulates the migration of vascular endothelial cells (44), which is necessary to promote normal external embryonic angiogenesis and embryonic cardiac development, and maintain the normal structure and integrity of the adult vasculature (45), and may play a key role in endothelial cell binding to integrins and/or other RGD receptors (46). As a TGF-β co-receptor and participates in the TGF-β/BMP signaling cascade that ultimately leads to the activation of Smad transcription factor (43): endothelial cells mediate the GDF2/BMP9 signaling pathway through Smad1 and regulate TGF-β signaling pathway (43) through Smad3. CD109 antigen can regulate negatively of TGF-β signaling in keratinocytes (47).

Compared with MH group, the most down-regulated proteins in FH group were prolactin inducer protein, lactoferrin, extracellular glycoprotein lacritin and lysozyme C. In mammals, prolactin (PRL) affects human ovarian function，PRL binds with its receptors to stimulate luteinizing hormone (LH) receptor generation; the combination of LH and its receptor can promote ovulation, luteum formation and progesterone and estrogen secretion. The downregulation of inflammation-promoting lactoferrin in cornea of FH group may be related to sex differences in some ocular inflammation. The extracellular glycoprotein lacritin regulates the secretion of lacrimal acinar cells. Lysozyme dissolves bacteria in body fluids or tissues via the monocytic macrophage system and enhances the activity of immune reagents.

The two most significant domain enrichment analyses were the C-terminal domain of fibrous collagen and collagen triple helix copies (20 copies), and the domain with high enrichment content was: Collagen triple helix copies (20 copies), fibrous collagen C-terminal domain, von-Willebrand factor C-domain, C1q domain, LRR RRM. RRMS are by far the most common RNA binding module, consisting of 80–90 amino acids, with a β-β-β-αβ topology (48). Over 10,000 RRMS have been identified, most of which play a role in post-transcriptional gene expression. About 0.5–1% of genes contain RRMS, often with multiple copies in the same polypeptide (49).

Comparative proteomic analysis of cornea from male and female patients with high myopia revealed increased expression of proteins associated with extracellular matrix and collagen I, III, V, and VIII in female patients, and the TGF-β/Smad pathway was an important pathway obtained from functional analysis, suggesting that extracellular matrix remodeling and collagen fiber synthesis may be more active in female patients’ cornea.

## Data availability statement

The mass spectrometry proteomics data have been deposited to the ProteomeXchange Consortium (https://proteomecentral.proteomexchange.org) via the iProX partner repository with the dataset identifier PXD054620.

## Ethics statement

The ethical committee of Peking Union Medical College Hospital (PUMCH) authorized all of the study’s experiments, which were conducted in accordance with the Declaration of Helsinki (ZS-3516). Prior to the collection of tissues, written and informed consent was obtained. All procedures were carried out in accordance with institutional and governmental regulations, and all human samples were de-identified before analysis.

## Author contributions

GC: Conceptualization, Data curation, Formal analysis, Investigation, Methodology, Software, Validation, Writing – original draft, Writing – review & editing. YD: Data curation, Investigation, Methodology, Software, Validation, Writing – review & editing. SY: Data curation, Investigation, Methodology, Software, Validation, Writing – review & editing. YC: Funding acquisition, Project administration, Resources, Supervision, Validation, Visualization, Writing – review & editing. YL: Conceptualization, Investigation, Project administration, Resources, Supervision, Validation, Visualization, Writing – review & editing. DC: Conceptualization, Funding acquisition, Investigation, Methodology, Project administration, Supervision, Validation, Visualization, Writing – review & editing.
